# The Application of Ultrasmall Gold Nanoparticles (2 nm) Functionalized with Doxorubicin in Three-Dimensional Normal and Glioblastoma Organoid Models of the Blood–Brain Barrier

**DOI:** 10.3390/molecules29112469

**Published:** 2024-05-24

**Authors:** Kathrin Kostka, Viktoriya Sokolova, Aya El-Taibany, Benedikt Kruse, Daniel Porada, Natalie Wolff, Oleg Prymak, Michael C. Seeds, Matthias Epple, Anthony J. Atala

**Affiliations:** 1 Inorganic Chemistry and Center for Nanointegration Duisburg-Essen (CENIDE), University of Duisburg-Essen, Universitaetsstr. 5-7, 45141 Essen, Germany; 2 Wake Forest Institute for Regenerative Medicine, Wake Forest School of Medicine, Winston-Salem, NC 27101, USA

**Keywords:** gold, nanoparticles, doxorubicin, organoids, glioblastoma, blood–brain barrier, drug delivery

## Abstract

Among brain tumors, glioblastoma (GBM) is very challenging to treat as chemotherapeutic drugs can only penetrate the brain to a limited extent due to the blood–brain barrier (BBB). Nanoparticles can be an attractive solution for the treatment of GBM as they can transport drugs across the BBB into the tumor. In this study, normal and GBM organoids comprising six brain cell types were developed and applied to study the uptake, BBB penetration, distribution, and efficacy of fluorescent, ultrasmall gold nanoparticles (AuTio-Dox-AF647s) conjugated with doxorubicin (Dox) and AlexaFluor-647-cadaverine (AF647) by confocal laser scanning microscopy (CLSM), using a mixture of dissolved doxorubicin and fluorescent AF647 molecules as a control. It was shown that the nanoparticles could easily penetrate the BBB and were found in normal and GBM organoids, while the dissolved Dox and AF647 molecules alone were unable to penetrate the BBB. Flow cytometry showed a reduction in glioblastoma cells after treatment with AuTio-Dox nanoparticles, as well as a higher uptake of these nanoparticles by GBM cells in the GBM model compared to astrocytes in the normal cell organoids. In summary, our results show that ultrasmall gold nanoparticles can serve as suitable carriers for the delivery of drugs into organoids to study BBB function.

## 1. Introduction

Glioblastoma (GBM) is the most common primary brain malignancy; it is highly aggressive, with a median survival time of about one year [1,2]. The major problems associated with GBM treatment are drug resistance and the restricted permeation of drugs across the blood–brain barrier (BBB) [3,4]. The selective permeability of the BBB leads to the ineffective treatment of GBM [5] as an intact BBB permits the entry of only about 1% of drugs into the brain tissue [6,7]. Therefore, new strategies to overcome these main obstacles are necessary. To overcome the complexity and drawbacks associated with in vivo models and to develop new platforms for screening new drugs, several novel and innovative 2D and 3D cell culture models for GBM have been proposed and implemented [8,9]. One approach that has been extensively investigated deals with the formation of 3D organoids [10]. These organoids are formed from different cell types without any solid supports, allowing the cells to form floating cell clusters with a spheroid-like architecture. Another 3D model is based on hydrogel scaffolds which are formed by soft, water-swollen materials, such as natural or synthetic cross-linked polymer chains, usually supplemented with other components like proteins of the extracellular matrix (ECM) [11]. Some of the most promising 3D models are organoids comprising aggregates of multiple cell types that represent the in vivo architecture of the tissue of origin [12,13]. Ultrasmall gold nanoparticles (with a diameter of around 2 nm) enable efficient transfer across biological barriers, including cellular and even nuclear membranes [14,15,16]. Furthermore, in contrast to gold nanoparticles bigger than 10 nm, they do not quench fluorescence [17], therefore, they can be visually monitored by confocal laser scanning microscopy (CLSM) if dye molecules are attached to their surfaces. Different kinds of molecules or therapeutic drugs can be covalently attached to the surfaces of ultrasmall gold nanoparticles by click chemistry [18,19]. Such nanoparticles have been successfully applied as delivery systems for biomolecules [20,21,22].

By combining established 3D GBM models and ultrasmall gold nanoparticles functionalized with doxorubicin and fluorescent AlexaFluor-647-cadaverine (AF647), we studied the anti-cancer effect of these nanoparticles in 3D BBB models, i.e., normal and GBM organoids. These organoids consisted of six different brain cell types; the astrocytes were replaced with U87-MG cells (glioblastoma cells) in case of the GBM model. Both the normal and GBM organoids formed an intact BBB with an endothelial cell surface and were able to serve as reproducible in vitro platforms to test therapeutic nanoparticles for GBM treatment. Doxorubicin is clinically applied in the treatment of GBM. However, its limited BBB permeability results in diminished bioactivity in GBM [23]. We developed ultrasmall gold nanoparticles conjugated with both doxorubicin and AF647 to improve the transport of doxorubicin across the BBB and investigated their cytotoxicity in normal and GBM models.

## 2. Results

**Figure 1:**
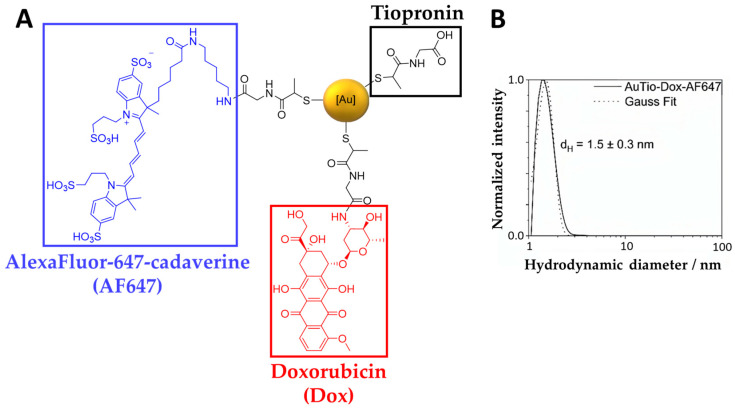
The chemical structures (shown schematically) of the ultrasmall gold nanoparticles carrying the fluorescent dye AF647 (left, blue), the cytostatic agent Dox (bottom, red), and the linker tiopronin (right, black) (**A**). Hydrodynamic particle size distribution as shown by DCS (**B**).

Ultrasmall gold nanoparticles were surface-functionalized by EDC/NHS, coupling them with doxorubicin alone or with doxorubicin and AF647 together for diagnostic (imaging) and therapeutic applications in 2D and in 3D cell culture models (organoids) [15,19]. The schematic structure of the AuTio-Dox-AF647s is shown in Figure 1A. Tiopronin (black) was the linker used to conjugate the AF647 (blue) and doxorubicin (red) to the nanoparticle surface. The colloid–chemical characterization is given in Figure 1. As in Sokolova et al. [24], no shifts in the characteristic fluorescence excitation and emission maxima of the conjugated doxorubicin and AF647 (AuTio-Dox-AF647s; *λ*_ex,Dox_ = 494 nm, *λ*_em,Dox_ = 522 nm, *λ*_ex,AF647_ = 647 nm, and *λ*_em,AF647_ = 666 nm) were observed (data not shown). A broad absorption band of the conjugated doxorubicin was found together with the absorption bands of AF647 in the UV–Vis spectrum, but no surface plasmon resonance was observed, as is expected for ultrasmall nanoparticles (data not shown). A hydrodynamic diameter of 1.5 ± 0.3 nm was determined by differential centrifugal sedimentation (DCS) to prove the ultrasmall character of the nanoparticles (Figure 1B). This measured size is underestimated due to the low overall density of the nanoparticles and the ligands on their surfaces; in addition, only the density of pure gold was used to compute the nanoparticle size. The actual size of the nanoparticles was not exactly 1.5 nm but was larger.

**Figure 2:**
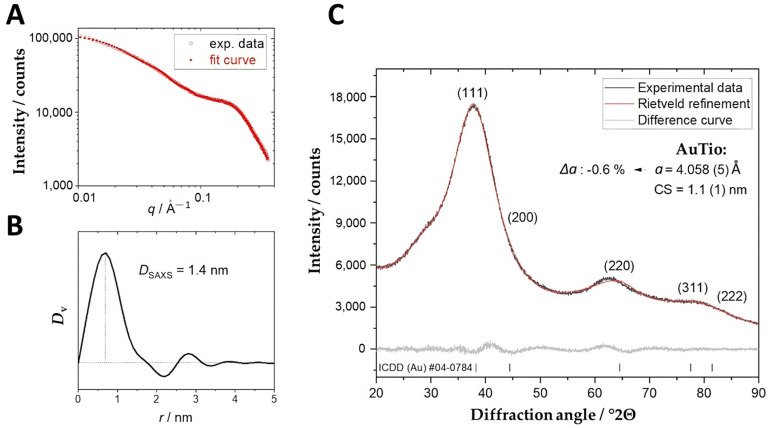
The results of a SAXS assessment of water-dispersed AuTio-Dox ultrasmall gold nanoparticles with the experimental data *I*(*q*) and model fit (**A**) and the volume-weighed particle size distribution *D*_v_ with the determined diameter *D*_SAXS_ (**B**). A representative X-ray powder diffractogram (**C**) with a Rietveld refinement (*R*_wp_ = 2.0) of AuTio nanoparticles with calculated lattice parameters (a) and crystallite size (CS). Numbers in parentheses indicate the Miller indices of the diffraction peaks of gold.

A small-angle X-ray scattering (SAXS) assessment of the ultrasmall gold nanoparticles (AuTio-Dox) showed a monomodal particle size distribution with an average gold core diameter of about 1.4 nm (Figure 2A). The determined size was in a good agreement with the DCS data (Figure 1B). X-ray powder diffraction (Figure 2B) showed very broad diffraction peaks of nanocrystalline gold without sharper peaks. This result agreed with the monodispersity found by DCS (Figure 1B) and SAXS (Figure 2A). The crystallite size of the AuTio nanoparticles was smaller than the particle size, so a polycrystalline (twinned) nature of the nanoparticles is likely. Furthermore, the AuTio nanoparticles had a slightly compressed unit cell (∆*a* = −0.6%) compared to microcrystalline Au (*a* = 4.078 ± 0.001 Å) (Figure 2C).

The full characterization data of the nanoparticles are shown in Table 1.

**Figure 3:**
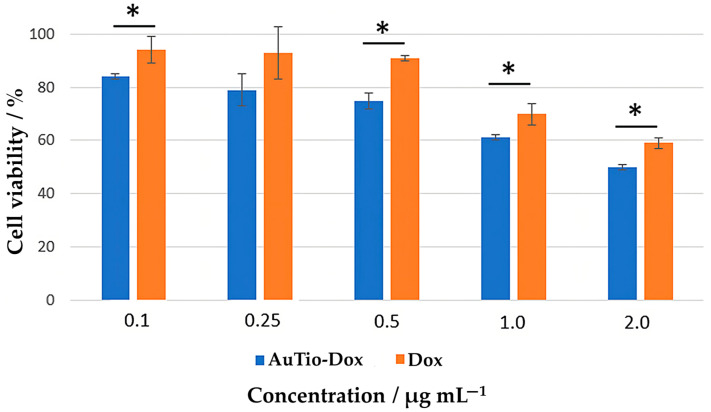
MTT assay of T98GBM cells incubated with AuTio-Dox nanoparticles (blue) and Dox molecules alone (orange) as a function of doxorubicin concentration. * = Significance: * *p* ≤ 0.1.

Initial investigations of the ultrasmall gold nanoparticles were carried out in 2D cell culture models. The cytotoxicity of the AuTio-Dox nanoparticles to the T98GBM cell line was evaluated with a viability assay (Figure 3). T98GBM cells were incubated with the AuTio-Dox nanoparticles for 24 h at different concentrations (0.1–2.0 µg mL^−1^). Dissolved doxorubicin at the same concentrations as the nanoparticles was used as a control.

A decrease in cell viability was observed with an increasing concentration of AuTio-Dox nanoparticles. At a concentration of 0.1 µg mL^−1^ of AuTio-Dox nanoparticles, the cell viability was about 80%, whereas at 2 µg mL^−1^, the viability decreased to 50%. With 0.1 µg mL^−1^ of doxorubicin, the cell viability was around 90%, and it dropped to about 60% at 2 µg mL^−1^. Obviously, the viability of the T98GBM cells was affected by doxorubicin in the same way whether it was present in the form of dissolved molecules or attached to nanoparticles.

**Figure 4:**
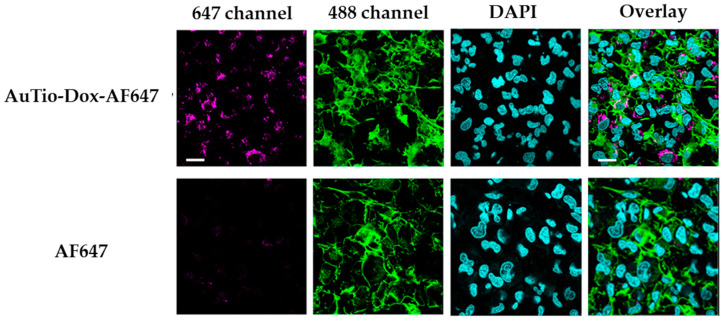
CLSM images of T98GBM cells incubated for 24 h with either fluorescent ultrasmall gold nanoparticles (AuTio-Dox-AF647s) or AF647 molecules alone. AF647 (magenta; AF647 channel), actin staining with AlexaFluor-488 phalloidin (green; AF488 channel), nuclear staining with DAPI (blue), and an overlay of all channels. Scale bars: 10 µm. The AuTio-Dox-AF647 nanoparticles can be detected as red, fluorescent dots in the cells (first row), whereas no fluorescence is visible after treatment with the AF647 molecules alone (second row).

The uptake of AuTio-Dox-AF647 nanoparticles and AF647 molecules alone into T98GBM cells (using a 2D cell culture model) was visualized by CLSM (Figure 4). The uptake percentage of the AuTio-Dox-AF647 nanoparticles was about 74%.

The AuTio-Dox-AF647 nanoparticles were taken up by the cells well, but the AF647 molecules alone did not enter the cells. This underscores the ability of ultrasmall nanoparticles to act as carriers for drug molecules.

A variety of 2D cell culture models, ranging from immortalized cells to 3D organoids, have been developed for modeling normal and GBM organoids [25]. Organoids have major advantages over other in vitro models as they resemble the in vivo architecture of the tissue of origin and can better reconstruct cell self-organization, differentiation, and cell–cell communication, thus mimicking the important features of the in vivo microenvironment. Normal and GBM organoids were developed from six human brain cell types, as previously described [26,27,28]. Astrocytes (or U87-MG cells for the GBM model), microglial cells, oligodendrocytes, and neurons were cultured for 48 h before an outer layer of pericytes and endothelial cells was added. The morphology of the organoids was not specifically considered. Care was taken to ensure that the organoids remained roughly spheroidal, did not fracture, and did not acquire distinct morphologies under the same conditions tested (normal vs. GBM organoids and organoids with various conjugated gold nanoparticles); therefore, specific surface areas or volumes were not measured.

**Figure 5:**
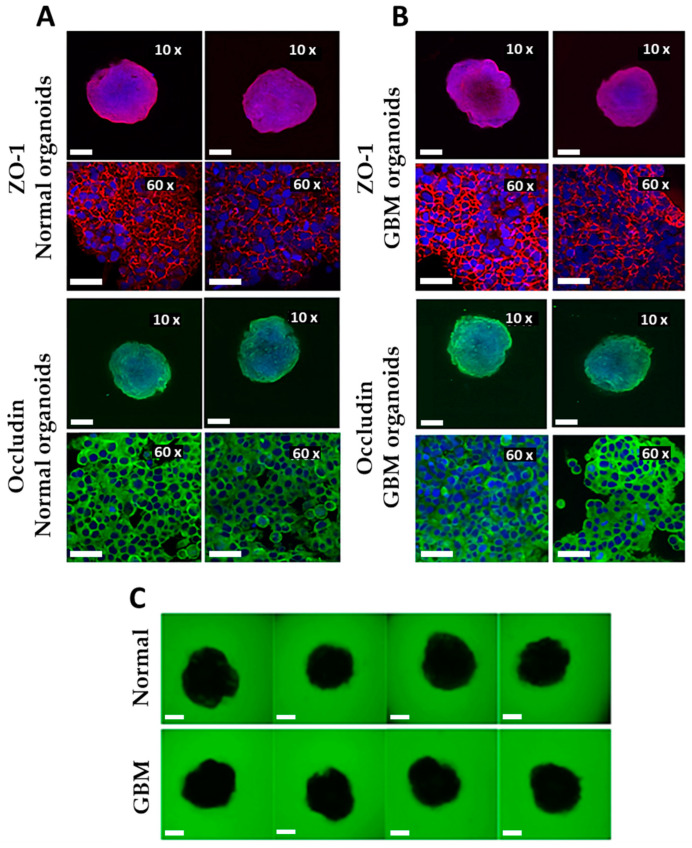
Characterization of normal and GBM organoids. Organoids were stained with either ZO-1 or occludin, and the nuclei were stained with DAPI for immunostaining ((**A**) normal organoids and (**B**) GBM organoids). A higher magnification (×60) was used to visualize the tight junctions (network structures) between endothelial cells. Scale bars: 200 μm (×10) and 50 μm (×60). Permeability was determined with fluorescent dextran (green) after 30 min of incubation (**C**). The BBB was intact, and no fluorescent signal was visible inside the organoids. Scale bars: 200 μm.

The organoids were characterized in terms of their tight junctions by immunostaining (Figure 5A,B) and the permeability of the BBB (permeability assay; Figure 5C,D).

The normal and GBM organoids were intact with good BBB integrity. Both organoid types showed complete ZO-1 and occludin borders (network structures; 60x magnification) around the endothelial cells on their surfaces. The BBB was also examined for permeability. This was demonstrated by the exclusion of fluorescent dextran (green). As shown in Figure 5C, no fluorescent dextran can be detected within the normal and GBM organoids, indicating the tightness of the BBB.

**Figure 6:**
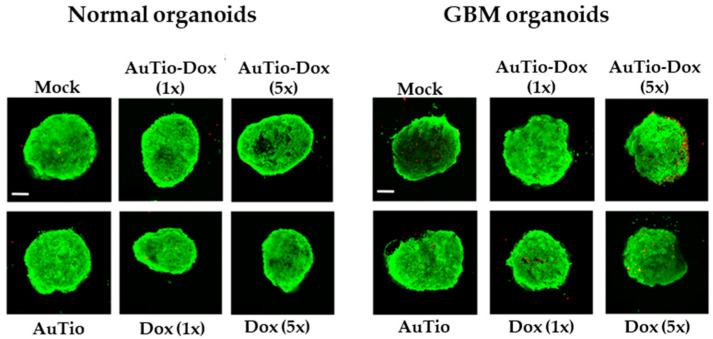
Live/dead assay. Living cells are shown in green, and dead cells are shown in red. Normal and GBM organoids after incubation with AuTio-Dox nanoparticles and doxorubicin molecules alone; (1×) corresponds to a normal concentration and (5×) corresponds to a five times higher concentration of AuTio-Dox (5×) or doxorubicin (5×), respectively. Scale bars: 200 µm.

The organoids were cultured for another 6 to 7 days before they were incubated with nanoparticles and control groups (mock, AuTio, and dissolved doxorubicin molecules) for 24 h before characterization. The nanoparticles were subsequently investigated in normal and GBM organoids as 3D cell culture models. First, a live/dead assay was performed with AuTio, AuTio-Dox (1× and 5× concentration, based on doxorubicin), and doxorubicin molecules alone at the same concentrations (Figure 6). For this, 7-day-old organoids were incubated for 24 h with nanoparticles and Dox molecules (control).

The live/dead assay showed that the gold nanoparticles, Au-Tio and AuTio-Dox, and Dox alone were not cytotoxic for the cells in the normal and GBM organoids based on the red, fluorescent cells (dead cells). A greater increase in the number of dead cells in the GBM organoids was measured following incubation with the AuTio-Dox nanoparticles at a five times higher concentration of doxorubicin (AuTio-Dox (5×)); however, incubation with Dox (5×) alone did not show any significant increase in the number of dead cells.

For uptake studies using CLSM, the concentration of doxorubicin in all gold nanoparticle dispersions was the same (Table 2) in order to achieve a better comparison between the nanoparticle types and their effectiveness in organoids after incubation. The number of doxorubicin molecules in each well is given in Table 2.

**Figure 7:**
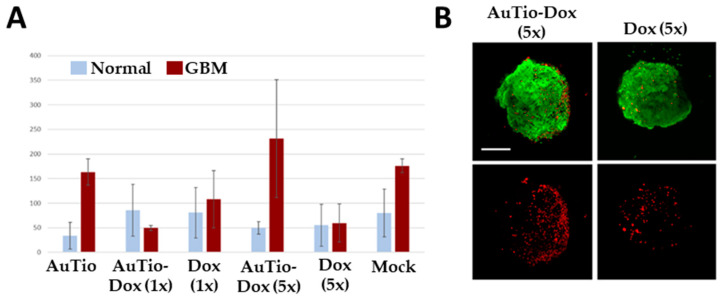
Live/dead assay. Average number of red dead cells calculated from five organoids (**A**). Determination of average number of red cells quantified inside brain organoids after uptake of AuTio-Dox nanoparticles and doxorubicin molecules alone after 24 h of incubation. Number of red cells was calculated and averaged from *n* = 5 organoids using FIJI. Representative images are shown for AuTio-Dox (5×) and Dox (5×) from CLSM (**B**). Scale bars: 200 µm.

For quantitative data, we counted the number of dead cells per organoid and summarized this information in Figure 7.

The average numbers of red dead cells in the normal and GBM organoids in Figure 7 were analyzed for their significance for each nanoparticle type and for the doxorubicin molecules alone. The greatest significance can be seen for the AuTio-Dox nanoparticles, and the lowest significance can be seen for the Dox (5×) molecules alone (Table 3).

The viability of the organoids after 24 h of incubation with the nanoparticles and doxorubicin molecules was estimated by measuring ATP production. There were no significant differences between the groups. This confirms that the viability of the organoids was not affected.

**Figure 8:**
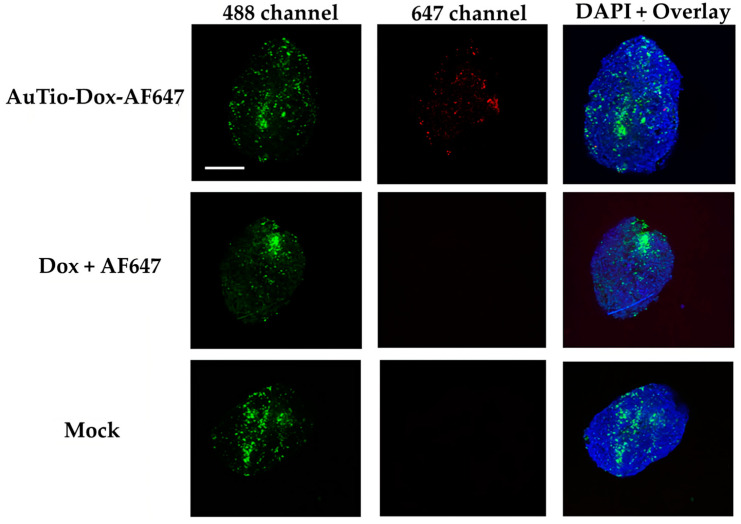
Uptake studies of the AuTio-Dox-AF647 nanoparticles and a mixture of dissolved doxorubicin and AF647 in normal organoids after 24 h of incubation. Astrocytes were fluorescently labeled with PKH-CellTracker (green; 488 channel). The AuTio-Dox-AF647 nanoparticles showed red fluorescence (647 channel; first row) in the organoids, while the mixture of doxorubicin and AF647 molecules showed no fluorescence (second row). Scale bars: 200 µm.

**Figure 9:**
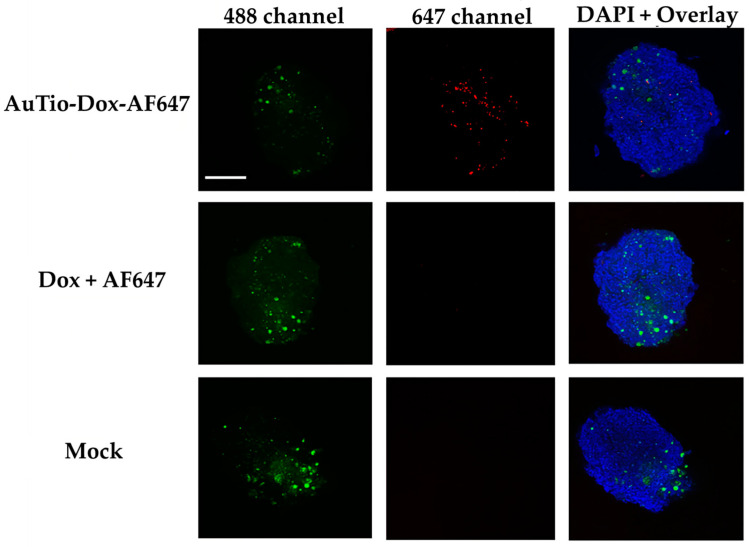
Uptake studies of AuTio-Dox-AF647s and dissolved doxorubicin and AF647 in GBM organoids after 24 h of incubation. U87-MG cells were fluorescently labeled with PKH-CellTracker (green; 488 channel). The nanoparticles showed red fluorescence. The AuTio-Dox-AF647 nanoparticles entered the organoid (red dots; 647 channel), whereas the doxorubicin and AF647 molecules alone showed no fluorescence (second row). Scale bars: 200 µm.

For uptake and penetration studies in the normal and GBM organoids, the AuTio-Dox-AF647 nanoparticles were used and incubated for 24 h. These nanoparticles carried AF647 and doxorubicin. As a control, a mixture of dissolved doxorubicin and AF647 at the same concentration as in the nanoparticles was applied. Astrocytes (normal organoids) and U87-MG cells (GBM organoids) were stained with green, fluorescent PKH-CellTracker (Figure 8 and Figure 9, respectively) before microscopy. PKH-CellTracker dyes are simultaneously cell tracker and proliferation dyes. As the organoids grow, the stained astrocytes and U87-MG cells divide and migrate to different parts of the organoid, yielding a diffuse localization. The cell tracker dye intensity is also diluted in daughter cells but remains detectable by fluorescent microscopy and flow cytometry. Overall, the images consistently yielded a heterogenous distribution and fluorescent intensity for the astrocytes and U87-MG cells.

It was shown that the AuTio-Dox-AF647 nanoparticles were taken up and distributed within the organoids (red dots in the 647 channel), while neither the dissolved doxorubicin nor AF647 were detected (Figure 8 and Figure 9). A particle analysis of the AuTio-Dox-AF647 nanoparticles inside the organoids was performed. There is no statistical significance (using two paired samples for means in a *t*-test) between normal and GBM organoids incubated with AuTio-Dox-AF647 nanoparticles. The normal and GBM organoids took up the same number of AuTioDox-AF647 nanoparticles. Table 4 shows the *p*-values from the *t*-test.

**Figure 10:**
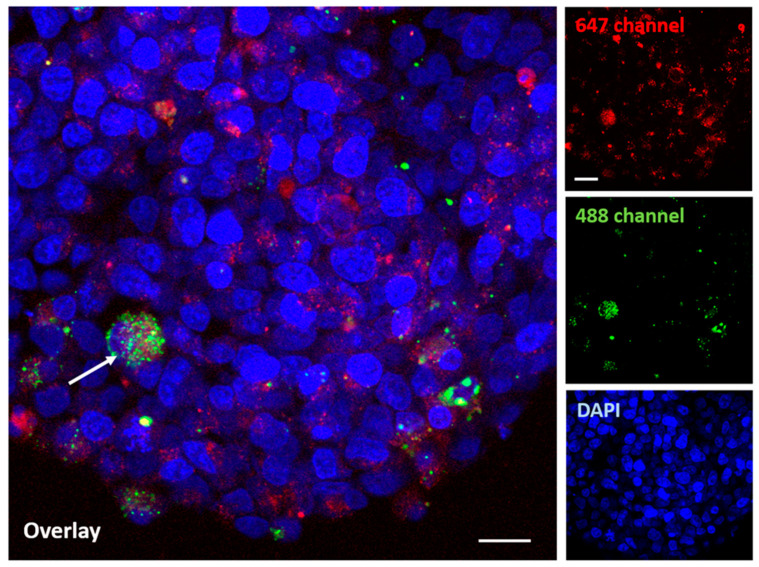
The uptake of AuTio-Dox-AF647s in a GBM organoid after 24 h at a higher magnification. The nanoparticles show a red fluorescence (647 channel), and the GBM organoid shows a green fluorescence (with arrow; labeled with PKH-CellTracker; green; 488 channel). A good distribution of the AuTio-Dox-AF647 nanoparticles in the organoid can be visualized. Scale bars: 10 µm.

Figure 10 shows the distribution of nanoparticles in a GBM organoid at a higher (60×) magnification.

Therefore, a co-localization of the AuTio-Dox-AF647 nanoparticles (red dots) and the green, fluorescent U87-MG cells is visible (white arrow).

**Figure 11:**
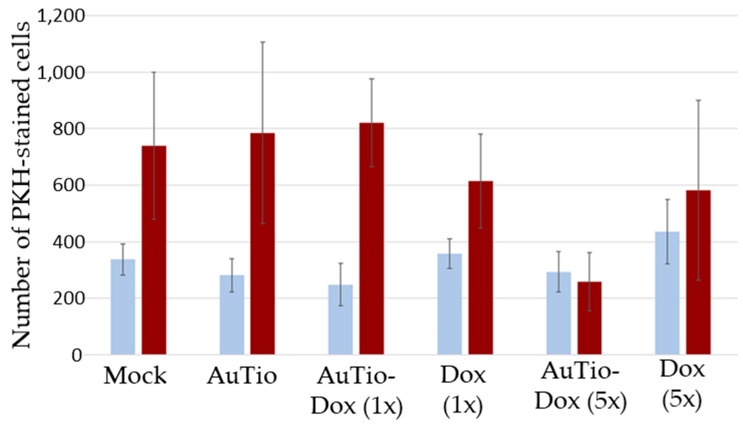
Number of PKH-CellTracker-stained cells (astrocytes in normal organoids and U87-MG cells in GBM organoids) quantified from five organoids using CLSM images of normal and GBM organoids. The determination of the number of PKH-CellTracker-stained cells quantified from inside the BBB organoids after the uptake of ultrasmall gold nanoparticles and doxorubicin molecules after 24 h of incubation. The average was calculated using the software FIJI.

To determine the anti-cancer effect of the AuTio-Dox nanoparticles in comparison to Dox molecules alone, the number of PKH-CellTracker-stained cells in the normal and GBM organoids was calculated (Figure 11).

No differences were observed in the number of fluorescing cells regarding the treatment in normal organoids, but a significant decrease in glioblastoma cells was observed in the 5x more concentrated AuTio-Dox nanoparticle group.

An FACS analysis confirmed the results obtained by CLSM that doxorubicin kills astrocytes when conjugated to nanoparticles but not when administered as doxorubicin molecules alone (not shown).

## 3. Discussion

These experiments were based on the hypothesis that conjugating a drug with limited BBB permeability to an ultrasmall gold nanoparticle would improve its delivery across the BBB. These data demonstrate proof of concept that such drug-conjugated nanoparticles do penetrate the BBB of a human 3D neurovascular organoid, implying drug delivery supported by the increased cell death of U87-MG cells relative to normal astrocytes when a high concentration of conjugated doxorubicin was used, whereas a concentrated doxorubicin solution alone had no effect. Our data further demonstrate that U87-MG cells can be incorporated into a 3D BBB organoid with no impact on the permeability or viability of cells within the organoid. Therefore, organoids and nanoparticles offer a platform for screening other drug candidates with activity against glioblastoma cells. The inclusion of other disease-related cell lines would expand the utility of the human 3D BBB organoid model and augment the use of 2D cell cultures and animal models, which often do not represent the cell-to-cell communication and tissue physiology seen in 3D human organoids.

The use of various cytostatic drugs such as 5-fluorouracil, paclitaxel, and doxorubicin in combination with nanoparticles has been tested for cancer treatment [26,27,28,29,30,31,32]. However, doxorubicin has shown significant limitations in terms of BBB penetration in the past, indicating the need for a suitable carrier system. Therefore, Lakkadwala et al. developed liposomal nanoparticles for the delivery of doxorubicin into GBM organoids which resulted in approximately 52% tumor cell death [33]. Niu et al. attached heparin-based nanoparticles loaded with doxorubicin to the surfaces of extracellular vesicles and showed that they could penetrate glioma tissue via receptor-mediated transcytosis, which significantly increased the efficacy of the antiglioma drugs [34]. Janjua et al. synthesized 30 nm silica nanoparticles with doxorubicin and tested them for the treatment of GBM. To achieve active targeting of GBM cells, the nanoparticles were additionally conjugated with lactoferrin, the receptors for which were overexpressed [35]. Ahmed et al. developed polymer–lipid hybrid nanoparticles for the delivery of doxorubicin to GBM cells and showed a sevenfold higher efficacy compared to free doxorubicin [36].

Ultrasmall gold nanoparticles were synthesized and applied as they are known to be bioinert and therefore have no cytostatic properties [37,38]. Due to their small size of a few nm, they can easily overcome the BBB [15,39]. Using EDC/NHS coupling, their surfaces were conjugated to not only doxorubicin molecules but also fluorescent AF647 molecules for imaging. This fluorescent labeling confirmed the successful uptake of the nanoparticles with the doxorubicin into the cells of the BBB-GBM organoid model, as demonstrated by CLSM. Accordingly, these metallic particles are also stable under biological conditions and can release their cargo within organoids. AuTio-Dox nanoparticles very efficiently penetrate the BBB (in organoids) and have the potential to increase the efficiency of doxorubicin in in vitro 3D models.

In general, these nanoparticles have the potential to transport other cancer drugs across the BBB to reach brain tumor sites. The nanoparticles can be used to kill cancer cells in GBM organoids and to achieve higher cell viability in normal organoids. Thus, a targeted application of these nanoparticles is possible.

Although this platform provides great promise for screening treatments for glioblastoma, there are limitations to this platform in its current form. For example, it is unclear whether a separate blood–brain tumor barrier (BBTB) is formed in this first model. The confocal microscopy images (Figure 8, Figure 9 and Figure 10) suggest that this may require the pre-formation of a tumor mass prior to its incorporation into the 3D BBB organoid or a longer maturation of the GBM organoids to establish a BBTB. Additionally, the differential cell division between normal and cancer cells for any of the constituent cells in the BBB organoid may impact the application of this concept. However, measures of the overall apparent size, morphology, and baseline impermeability of the normal, healthy astrocyte BBB organoids and the GBM organoids were very similar, suggesting that these and variations of these 3D human organoids can provide a successful platform for drug screening for a variety of cell-based neurological diseases or similar organoids of other tissue types.

## 4. Materials and Methods

### 4.1. Reagents

To prepare tetrachloroauric acid (HAuCl_4_), elemental gold (≥99%) was dissolved in aqua regia. N-(2-mercaptopropionyl)glycine (Tiopronin; ≥98%), sodium borohydride (NaBH_4_; >96%), N-hydroxysuccinimide (NHS; 98%), and deuterium oxide (D_2_O; 99%) were obtained from Sigma-Aldrich (Steinheim, Germany). Doxorubicin hydrochloride (Dox; >99%) was obtained from Alfa Aesar (Kandel, Germany). AlexaFluor-647-cadaverine (AF647-cadaverine; 99%), (3-(4,5-dimethylthiazol-2-yl)-2,5-diphenyltetrazolium bromide (MTT), phosphate-buffered saline (PBS), AlexaFluor-488 phalloidin (actin staining), and DAPI (nuclear staining) were obtained from ThermoFisher Scientific (Darmstadt, Germany). 3.7% paraformaldehyde solution (PFA; p.a.) was obtained from Merck (Darmstadt, Germany). Dimethyl sulfoxide (DMSO; >99.5%) and 1-ethyl-3-(3-dimethylaminopropyl)carbodiimide-hydrochloride (EDC·HCl; >99%) were purchased from Carl Roth (Karlsruhe, Germany).

Eagle’s Minimum Essential Medium (EMEM) was obtained from the American Type Culture Collection (ATCC; Manassas, VA, USA). DMEM/F-12 (supplemented with 10% heat-inactivated Fetal Bovine Serum (FBS) and 1% by volume of Primocin, cat# ant-pm-05 (antibiotic–antimycotic)) from InvivoGen (San Diego, CA, USA) fluorescent dextran (Oregon Green™ 488; 70 kD, anionic, lysine-fixable), 2 μM calcein AM (green: living cells) and 4 μM ethidium homodimer-1 (red: dead cells) were obtained from ThermoFisher Scientific (Waltham, MA, USA). Neural maintenance-XF medium was obtained from Nexcelom Bioscience (Lawrence, KS, USA). An astrocyte medium (AM) and an endothelial cell growth medium (EGM) were purchased from Lonza (Basel, Schweiz). Rat tail collagen-I was purchased from Corning (New York, NY, USA). L-glutamine, non-essential amino acids, N2 supplement, and essential amino acids were obtained from Life Technologies (Carlsbad, CA, USA). PKH-CellTracker was purchased from MilliporeSigma (St. Louis, MO, USA). CellTiter-Glo substrate and CellTiter-Glo buffer were purchased from Promega Life Sciences (Madison, MA, USA). Tween-20, anti-human ZO-1 (MABT339), ReNcell NSC maintenance medium, biotin, cAMP, and accutase were obtained from Sigma-Aldrich (St. Louis, MO, USA). StemPro neural supplement, PDGF-AA, PDGF-AB, NT3, GM-CSF, and IL-34 were purchased from PeproTech (Cranbury, NJ, USA). Anti-human occludin (ab216327), goat anti-rabbit IgG H&L AlexaFluor-488 (ab150077), and goat anti-mouse IgG H&L AlexaFluor-594 (ab150120) were purchased from Abcam (Cambridge, UK).

For all nanoparticle syntheses and purifications, ultrapure water with a specific resistivity of 18.2 MΩ, prepared using a ELGA purelab ultra instrument (Runcorn, UK), was used unless otherwise noted. All glassware was cleaned with boiling aqua regia and thoroughly washed with water before nanoparticle synthesis.

### 4.2. Instruments

After synthesis, the nanoparticles were washed several times in ultrapure water for 20 min at 4000 rpm with centrifugal ultra-15 spin filters (Amicon*^®^* Ultra, 10 kDa, Merck; Tullagreen, Ireland) to remove DMSO from the dispersion. UV–Vis spectra were recorded on a Genesis 50 instrument (Agilent Technologies; Santa Clara, CA, USA) with a quartz cuvette. A sucrose gradient capped with 0.5 mL dodecane was used for differential centrifugal sedimentation (DCS), which was performed using a DC24000 UHR instrument (CPS Instruments; Prairieville, LA, USA). Dispersed PVC standard particles (483 nm; from CPS) were used for calibration. To determine the gold content of the samples, atomic absorption spectroscopy (AAS) was conducted with an Electron M-Series spectrometer (Thermo Electron; Waltham, MA, USA). Each AAS sample was prepared with 10 µL of the nanoparticle dispersion dissolved in 990 µL of aqua regia and then diluted with 3 mL of water before analysis. For X-ray powder diffraction (XRD), the particles were first lyophilized with a Christ Alpha 2–4 LSC instrument (Martin Christ GmbH; Osterode am Harz, Germany). XRD was used to provide information about the crystallinity of the ultrasmall gold nanoparticles and to identify a potential change in the unit cell size in relation to the ultrasmall particle size. The XRD measurements were performed with Cu Kα radiation (λ = 1.54 Å; U = 40 kV; I = 40 mA) using a D8 Advance diffractometer (Bruker AXS GmbH; Karlsruhe, Germany) in reflection mode. For this, freeze-dried AuTio nanoparticles were placed on a silicon single-crystal sample holder to minimize scattering and then measured from 20 to 90° 2Θ with a step size of 0.02° and a counting time of 20 s, resulting in a total measurement time of 13.6 h. For the qualitative phase analysis, the Bruker software Diffrac, Suite EVA V1.2, was used with the cubic Au pattern (#04-0784) from the ICDD database. To calculate the lattice parameters and the average crystallite size (CS) from diffraction peak broadening, a quantitative Rietveld refinement was performed with the Bruker software TOPAS 5.0. Small-angle X-ray scattering (SAXS) was used to investigate the dispersity of gold nanoparticles and to determine their size. The SAXS measurements were carried out with a scanning line detector (1D) on a Panalytical Empyrean diffractometer (Malvern Panalytical; Almelo, The Netherlands) in transmission mode with a ScatterX-78 evacuated beam path (1∙10*^−^*^2^ mbar), Cu Kα radiation (λ = 1.54 Å, U = 40 kV, and I = 40 mA), and a sample-to-detector distance of 240 mm. Three different types of ultrasmall gold nanoparticles (colloidal dispersions of AuTio, AuTio-Dox, and AuTio-Dox-AF647 nanoparticles in water) were poured into glass capillaries (length—80 mm; outer diameter—1 mm; wall thickness—0.01 mm) and measured in the 2Θ range from −0.15° to 5.00° at a step size of 0.01° and with a total measurement time of 21 min. For background correction, the same capillary was filled with water and measured again. The SAXS analysis was performed with Panalytical EasySAXS software 2.0, assuming a polydisperse system of spheres. From the measured scattering intensity, I(q), the volume-weighted size distribution (D_V_) was determined.

### 4.3. Synthesis of Functionalized Gold Nanoparticles

The synthesis of the functionalized gold nanoparticles (AuTio, AuTio-Dox, and AuTio-Dox-AF647) was carried out as reported in Sokolova et al. [24]. In order to obtain a high conjugation of doxorubicin and AF647 dye on the nanoparticles, a synthesis route using tiopronine-stabilized (adapted Brust–Schiffrin synthesis) ultrasmall gold nanoparticles (with a core diameter of 2 nm) was chosen. They were functionalized in the next step via EDC/NHS coupling. AuTio nanoparticles were synthesized from a HAuCl4 solution (127 µmol, 25 mg Au), stabilized with tiopronine (509 µmol, 83 mg), and reduced using a NaBH_4_ solution (200 mM, 7.6 g L*^−^*^1^). To avoid agglomeration, 5 mL of NaOH (0.1 M) was added, giving the particles a negative charge due to ionization. To prepare AuTio-Dox nanoparticles, doxorubicin (1.2 mL, 2.0 µmol) was added to the AuTio nanoparticle dispersion. The doxorubicin- and dye-conjugated nanoparticles (AuTio-Dox-AF647) were obtained after adding doxorubicin (0.6 mL, 1 µmol) and AlexaFluor647-cadaverine (0.05 mL, 0.05 µmol) simultaneously to the AuTio nanoparticle dispersion. At t = 0, 30, 60, and 90 min of reaction time, 1-ethyl-3-carbodiimide (EDC·HCl, 1.3 mg, 6.8 µmol) and N-hydroxysuccinimide (NHS, 1.6 mg, 13.9 µmol) were added, respectively. To remove unreacted educts and by-products, all functionalized nanoparticles were purified by spin filtration by washing them with water until no fluorescence was observable in the permeate. The concentrated gold nanoparticle dispersion was recovered from the spin filter by sampling with a pipette (~350 µL).

The total number of tiopronin molecules attached to the surface of each nanoparticle was determined by NMR spectroscopy. The concentrations of the conjugated doxorubicin and AF647 were determined by UV–Vis spectroscopy, as reported earlier [22]. The gold concentration in a given dispersion of nanoparticles (AuTio, AuTio-Dox, and AuTio-Dox-AF647) was determined by AAS. Based on an average particle diameter of 2 nm (particles were assumed to be spheres), as determined earlier by HRTEM [22] and from the AAS data, the gold nanoparticle concentration was calculated. The concentration of attached molecules was calculated by UV–Vis spectroscopy using previously determined calibration curves. Subsequently, the ratio of the molecule concentration (tiopronin, doxorubicin, and AF647) and the gold nanoparticle concentration equaled the number of conjugated molecules on each nanoparticle.

### 4.4. Cultivation of Organoids

The organoids were prepared according to the protocols published by Nzou et al. [25,40]. The standard organoids contained six brain cell types, i.e., 30% primary human brain microvascular endothelial cells (HBMECs), 15% primary human brain vascular pericytes (HBVPs), 15% primary human astrocytes (HAs), 5% human iPSC-derived microglial cells (HMs), 15% human iPSC-derived oligodendrocyte progenitor cells (HOs), and 20% ReNcell CX human neural progenitor cells (RNs), with a total of approximately 2000 cells per organoid. For the GBM organoids, all steps were identical aside from the fact that the full proportion of astrocytes (15% of total) was replaced by U87-MG cells. In some organoids, both astrocytes and U87-MG cells were stained with PKH-CellTracker prior to their incorporation into the organoids. Plates with these stained cells were used to assess the nanoparticle uptake. Plates with unstained U87-MG cells and astrocytes were used for the ATP assays.

In particular, primary human brain microvascular endothelial cells (HBMECs) were grown in flasks coated with an attachment factor using complete classic media supplemented with a culture boost and an attachment factor according to the manufacturer’s instructions. Primary human brain microvascular pericytes (HBVPs) were expanded inside attachment-factor-coated vessels and cultured in a pericyte medium supplemented with 2% FBS, a pericyte growth supplement, and penicillin–streptomycin according to the manufacturer’s instructions under normal growth conditions (5% CO_2_ and 37 °C). Human astrocytes (HAs) were cultured under normal growth conditions in an astrocyte medium (AM) containing 2% FBS, an astrocyte growth supplement, and penicillin–streptomycin. Human iPSC-derived oligodendrocyte progenitor cells (HOs) and human iPSC-derived microglia (HMs) were cultured under normal growth conditions in DMEM/F-12 with the addition of supplements. The human iPSC-derived oligodendrocyte media contained DMEM/F12 with HEPES, L-glutamine (2 mM), a non-essential amino acids solution (100x), stemPro neural supplement, PDGF-AA (10 ng μL*^−^*^1^), PDGF-AB (10 ng μL*^−^*^1^), NT3 (10 ng μL*^−^*^1^), biotin (100 ng μL*^−^*^1^), and cAMP (5 µM mL*^−^*^1^). The human iPSC-derived microglia medium contained DMEM/F-12, N2 supplement (1x), essential amino acids (0.5x), L-glutamine (2 mM), GM-CSF (100 ng mL*^−^*^1^), and IL-34 (50 ng mL*^−^*^1^). The ReNcell CX human neural progenitor cell line (RN) was cultured in flasks coated with laminin 20 µg mL*^−^*^1^ and ReNcell NSC maintenance medium containing recombinant human FGF2 (20 ng mL*^−^*^1^) and recombinant human EGF (20 ng mL*^−^*^1^). The U87-MG cells were cultured beforehand in uncoated T-75 flasks under normal growth conditions in a pre-warmed DMEM/F-12 medium.

To make organoids, the cells were harvested using accutase. Organoids containing HAs, HMs, HOs, and RNs or U87-MG cells were seeded according to the ratio given above in 96-well round-bottom ultra-low attachment plates in 50% astrocyte basal medium (AM) without an astrocyte growth supplement and 50% neural maintenance-XF medium under normal growth conditions. The media mixture was supplemented with 5% heat-inactivated FBS and 10 ng μL*^−^*^1^ rat tail collagen-I to coat the neural–glial organoid. Then, 24 h later, HBMECs and HBVPs were harvested and added in ratios of 30% HBMECs and 20% HBVPs, respectively, in a mixture of media containing 50% astrocyte basal media (AM) without supplements and 50% endothelial cell growth basal medium (EGM) supplemented with 2% FBS, 0.04% hydrocortisone, 0.01% R3-IGF-1, 0.1% ascorbic acid, 0.1% heparin, and 1 µL mL*^−^*^1^ of GA-1000 (30 mg mL*^−^*^1^ of gentamicin and 15 µg mL*^−^*^1^ of amphotericin) under normal growth conditions. For the maintenance of organoids, an organoid maintenance medium was used which was composed of a mixture of 60% neural maintenance-XF medium, 20% AM, and 20% EGM with supplements. The organoids were then allowed to mature for 48 h and placed into 96-well ultra-low attachment plates for long-term cultivation. The organoids were used in the experiments after 7 days of cultivation. The medium was exchanged every other day until the experiments were conducted.

### 4.5. MTT Test in 2D Cell Culture Model (T98GBM Cell Line)

T98GBM cells were seeded in a 24-well plate at a cell density of 50,000 cells per well and cultured with 0.5 mL of EMEM overnight at 37 °C in a 5% CO_2_ atmosphere. The next day, the cells were incubated with AuTio-Dox nanoparticles and doxorubicin molecules at different concentrations of doxorubicin (0.1 µg mL*^−^*^1^; 0.25 µg mL*^−^*^1^; 0.5 µg mL*^−^*^1^; 1.0 µg mL*^−^*^1^, and 2.0 µg mL^−1^) for 24 h. After incubation, the cells were washed twice with PBS to remove excess nanoparticles and dead cells. Then, 500 µL of the MTT reagent (1 mg mL*^−^*^1^ in EMEM) was added to the cells, which were then incubated for 1 h at 37 °C under a 5% CO_2_ atmosphere. Then, the MTT medium was replaced by 300 µL of DMSO, and the cells were incubated for another 30 min at room temperature. Finally, the obtained solution was transferred to a 96-well plate, and the absorbance was measured at λ = 570 nm using a multiscan FC plate reader.

### 4.6. Uptake Studies of Fluorescent AuTio-Dox-AF647 Nanoparticles by T98GBM Cells Followed by CLSM

The T98GBM cells (50,000 cells per well) were incubated in an 8-well plate with fluorescent AuTio-Dox-AF647 nanoparticles for 24 h at 37 °C in a 5% CO_2_ atmosphere. The cells were fixed for 15 min with 3.7% PFA and then washed three times with PBS. Subsequently, the cells were incubated for 20 min with AlexaFluor-488 phalloidin for actin staining and for 15 min with a DAPI solution for nuclear staining. Then cells were washed with PBS and imaged by CLSM.

### 4.7. Immunostaining

After 7 days of cultivation, eight normal and eight GBM organoids were collected, washed three times with cold DPBS, and fixed overnight at 4 °C with 4% PFA. Then, the organoids were washed three times with DPBS, permeabilized with 0.1% Tween-20 for 15 min, and washed again. The organoids were blocked for 1 h at room temperature with Dako Protein Block Solution, washed, and incubated overnight at 4 °C with anti-human occludin and anti-human ZO-1 (primary antibodies) at a ratio of 1:200 in a Dako antibody diluent. The organoids were washed three times with DPBS and incubated with goat anti-rabbit IgG H&L AlexaFluor-488 and goat anti-mouse IgG H&L AlexaFluor-594 (secondary antibodies) at a ratio of 1:1000 for 1 h at room temperature. At least three organoids of each group were examined using CLSM for each stain (using 10× and 60× magnifications at the same laser intensity for comparison). Finally, 4–7 slices were captured, and z-stacks were performed.

### 4.8. Permeability Assay

Normal and GBM organoids were incubated with green, fluorescent dextran at a concentration of 50 µg mL*^−^*^1^ for 30 min. The organoids were moved from their media containing fluorescent dextran without washing to confocal dishes for examination by CLSM. z-stacks of the organoids were recorded, and z-projection images were created. The mean fluorescent intensity of the green fluorescence inside and outside the organoids was quantified with the program Image [41]. The relative permeability of an organoid was calculated as a ratio of the inside/outside mean fluorescent intensities. The results were compared between different groups and plotted as percent changes in the relative permeability.

### 4.9. Live/Dead Assay in Organoids

Cell viability was evaluated with a Molecular Probes Live/Dead cell reagent system containing 2 μM calcein AM and 4 μM ethidium homodimer-1. The organoids were incubated for 24 h with AuTio-Dox nanoparticles and doxorubicin alone (1× and 5× concentrations) at 37 °C under 5% CO_2_ conditions. After the organoids were washed three times with PBS, the organoids were incubated at room temperature for 10 min in an organoid medium with the live/dead cell reagent. After they were washed three times with PBS, the organoids were imaged by CLSM. The quantification of dead cells (red) was performed with the program ImageJ [41].

### 4.10. ATP Production

The viability of the organoids was determined as described earlier [25]. In detail, the organoids were transferred into an opaque-walled 96-well plate, and 100 µL of organoid medium was added to each well. Cell Titer-Glo reagent was thawed overnight and brought to room temperature before its use. The CellTiter-Glo reagent was prepared by mixing a CellTiter-Glo substrate and CellTiter-Glo buffer according to the manufacturer’s instructions. Then, 100 µL of CellTiter-Glo reagent was added to each well. Cell lysis was induced via strong mixing in an orbital shaker. The plate was first incubated at room temperature for 10 min to stabilize the luminescent signal. After this time, the organoids were incubated at room temperature for 30 min on an orbital shaker protected from light. Luminescence was measured with a GloMax Navigator plate reader. Background luminescence from media without organoids was then subtracted from the samples.

### 4.11. Uptake Studies of Fluorescent AuTio-Dox-AF647 Nanoparticles in Normal and GBM Organoids by CLSM

The previously prepared organoids (5 normal organoids and 5 GBM organoids) were incubated for 24 h with AuTio-Dox-AF647 nanoparticles and a mixture of AF647 and doxorubicin molecules. Then, the organoids were collected in a 1.5 mL tube, washed twice with PBS, and fixed with 3.7% PFA overnight at 4 ℃ and washed with PBS. For nuclear staining, the organoids were stained with DAPI (1:1000) for 10 min, washed again twice with PBS, and examined by CLSM.

### 4.12. Statistics

Data are expressed as the average ± standard deviation of the mean for each group. Student’s *t*-test was used to compare groups; *p*-values below 0.05 were considered significant.

## 5. Conclusions

The application of fluorescent ultra-small gold nanoparticles loaded with doxorubicin led to the uptake of the chemotherapeutic agent doxorubicin into normal and GBM organoids, resulting in the death of GBM cells. This was observed in a live/dead assay (Figure 6) in which normal and GBM organoids were treated with the nanoparticles under the same conditions. At a high concentration of AuTio-Dox nanoparticles, many dead cells were observed in the GBM organoids in contrast to the normal organoids. The high concentration of dissolved doxorubicin alone also resulted in fewer dead cells (Figure 7B). These observations confirmed the results of the 2D model with an MTT assay (Figure 3). There, in contrast to the dissolved doxorubicin molecules, the use of AuTio-Dox nanoparticles showed reduced cell viability. However, the particles were not only loaded with doxorubicin but also with AF647 in order to visualize their uptake into the organoids by the BBB. A good distribution in the organoids was observed by CLSM (Figure 8, Figure 9 and Figure 10), with the red, fluorescent AF647 molecules visible on the surfaces of the nanoparticles. Thus, doxorubicin was also successfully transported into the organoids via the BBB and subsequently distributed in the organoids. Control experiments showed that a mixture of AF647 and doxorubicin molecules alone could not pass the BBB (Figure 8 and Figure 9) and therefore could not enter the organoids.

## Figures and Tables

**Table 1:** Properties of all prepared ultrasmall gold nanoparticles, including concentrations used in biological experiments.

Parameters	AuTio	AuTio-Dox	AuTio-Dox-AF647
Solid core diameter, nm	2	2	2
Hydrodynamic diameter determined by DCS, nm	1.7 ± 0.5	1.7 ± 0.4	1.5 ± 0.3
Diameter (Au core) determined by SAXS, nm	1.3 ± 0.3	1.4 ± 0.4	1.6 ± 0.5
Crystallite size determined by XRD, nm	1.1 ± 0.1	-	-
Gold concentration in sample, mol L^−1^	5.08 × 10^−3^	3.60 × 10^−4^	5.08 × 10^−3^
Gold concentration in sample, g L^−1^	1.00	0.07	1.00
Particle concentration in sample, mol L^−1^	2.05 × 10^−5^	1.46 × 10^−6^	2.05 × 10^−5^
Particle concentration in sample, L^−1^	1.24 × 10^19^	8.78 × 10^17^	1.24 × 10^19^
Concentration of doxorubicin, mol L^−1^	-	2.67 × 10^−5^	2.65 × 10^−5^
Concentration of doxorubicin, mg mL^−1^	-	1.15 × 10^−2^	1.14 × 10^−3^
Doxorubicin molecules per particle	-	18.3	1.3
Concentration of AF647, mol L^−1^	-	-	1.22 × 10^−6^
Concentration of AF647, mg mL^−1^	-	-	1.12 × 10^−3^
AF647 molecules per particle	-	-	0.1

**Table 2:** Addition of gold nanoparticles and corresponding control groups to normal and GBM organoids.

Parameters	AuTio	AuTio-Dox	Dox	AuTio- Dox-AF647	Dox and AF647
*c* (Au stock dispersion), g L^−1^	1.000	0.071	-	1.000	-
*V* (medium) per well, µL	100	100	100	100	100
*V* (Au stock dispersion) well, µL	8	8	8	8	8
*m* (Au) per well, µg	8	0.568	-	8	-
*c* (Au) in the well, g L^−1^	0.07	0.006	-	0.07	-
Particle molar concentration (*d* = 2 nm) in the well, mol L^−1^	1.52 × 10^−6^	1.08 × 10^−7^	-	1.52 × 10^−6^	-
Particle number concentration (*d* = 2 nm) in well, L^−1^	9.16 × 10^17^	6.50 × 10^16^	-	9.16 × 10^17^	-
Particle number (*d* = 2 nm) in the well	9.89 × 10^13^	7.03 × 10^12^	-	9.89 × 10^13^	-
Molecules in each well	-	1.29 × 10^14^ (Dox)	1.29 × 10^14^ (Dox)	5.87 × 10^12^ (AF647)/ 1.29 × 10^14^ (Dox)	5.87 × 10^12^ (AF647)/1.29 × 10^14^ (Dox)

**Table 3:** *p*-values from a *t*-test: two samples paired for means.

Particle Type/ Doxorubicin Molecules	One-Sided	Two-Sided
AuTio nanoparticles	0.27	0.54
AuTio-Dox (1×) nanoparticles	0.04	0.09
Dox (1×) molecules	0.17	0.34
AuTio-Dox (5×) nanoparticles	0.08	0.16
Dox (5×) molecules	0.48	0.96
Mock (control group)	0.36	0.72

**Table 4:** *p*-values from *t*-test: two samples paired for means.

Nanoparticle Uptake	One-Sided	Two-Sided
Normal and GBM organoids	0.42	0.84

## Data Availability

Data are available from the authors upon request.
